# Supplementary material for: Facile Synthesis of Bio-Antimicrobials with “Smart” Triiodides
Source: Molecules. 2021 Jun 10;26(12):3553. doi: 10.3390/molecules26123553 (PMC8230494; doi:10.3390/molecules26123553)
Supplement: Supplementary file 1 [file molecules-26-03553-s001.zip › molecules-1199471-supplementary/Supplementary Table S1 FTIR all.pdf]

Supplementary Table S1. FTIR analysis [cm^{-1}] AV-PVP-Sage-I_2 (1), AV-PVP-Sage (2), Sage extract [14], AV-PVP-I_2 (3) [31], AV-PVP-NaI (4) [31], PVP pure (5) [74], [59] (cm^{-1}).

	ν_1 (O–H)*$_s$<br>ν_2 (O–H)*$_a$	ν (C–H)$_a$	ν (CH$_2$)$_{a,s}$<br>chain	ν (C=O)$_s$<br>or C–N bending	δ (C–H)$_a$<br>ν_3 (H–O–H)*<br>ν (C=C)$_{s,}$ aromat<br>C–N-C stretch K30	C–H bending, in plane<br>δ (C–C)$_s$?	δ (C–H)<br>C–N stretch<br>C–N stretch	δ (C–C)<br>CH$_2$<br>rock	ν (C–O)	ν (CH–CH)<br>ν (O–H)*
1,2<br>Abs	3231s,br<br>3027 m,sh ?	2952w	2915w,sym<br>2360s<br>2161m<br>2023m	1637s,br	1500m,br,sh<br>1463C=O	1348w<br>1364C=O	1286w	-	1230m,br<br>1176w<br>1105w<br>1067m<br>973w	897m
[14]	3248s,br<br>O-H<br>Phenolic<br>Or COOH	2929m,sh<br>CH$_2$	2162w<br>2026w	1585vs	-	1374s	1258m	-	1103s,sh<br>1034s<br>1007s	870w<br>817w<br>736w<br>695w
[20]	3406<br>O-H<br>Phenolic	2976<br>CH$_3$ as<br>2931 C-H		1653 C=C in flavonoids, terpenoids and C=O stretching proteins or amide	-	1464<br>N-H<br>from<br>proteins<br><br>1393<br>CN-<br>aromati	-	-	1094 carbonyl –C-O-C- or –C-O- stretching of amide linkages<br>1048<br>carbohydrates	879 CH2 in ethanol

						c amino<br>groups				
3	3397s,br<br>3320s,br<br>3229s,br<br>3009m	2952m	2919w<br>2837w<br>2359w<br>2334w<br>2203w<br>2024w	2146w,combination<br>1650m,br<br>1403w(COO- sym<br>stretch)	1472w<br>1498w	1348w<br>1361w	1286w<br>1291w<br>CH2wag	1013s	1245m<br>1118m<br>1106w<br>1066w<br>913m	932m<br>**822m**<br>**δ (CH_2)**
4	3669s<br>3397s,br<br>3320s,br<br>3229s,br<br>3009m	2952m	2920w<br>2843w<br>2359w<br>2334w<br>2203w<br>2024w	2146w,combination<br>1648m,br<br>1406w(COO-<br>symstretch)	1474w<br>1498w	1395w<br>1363w	1286w<br>1292w<br>CH_2wag	1013s	1250m<br>1114w<br>1106w<br>1066w<br>904m	924w<br>**839m**<br>**δ (CH_2)**
5	3396*$_a$	2950s	2922<br>2349	1646s<br>1660 PVPK30 for<br>C=O	-?1470<br>1444K30	1373s	1286m<br>CH_2wag<br>1292K30	1017w<br>C-C	-	933w C-C<br>**843w**<br>**δ (CH_2)**
[59]	3746(1)*$_s$<br>3418(2)*$_a$	2951(3)	2905(4)	2863(5)<br>2777(6)*<br>2733(7)*	1443<br>1642(13)*	1360(17)	1287(18)		1243(19)<br>1133(20)<br>1092(21)<br>1022(22)<br>911(23)	844(24)<br>549(26)*<br>628(25)*

* Signals related to vibrational modes of hydroxyl groups due to hydration in the compound B. Numbers in brackets indicate the signal numbers used in Figure 4. ν = vibrational stretching, δ = deformation, s = symmetric, a = asymmetric. - = peak is not available.
