# Supplementary figures and images for: Facile Synthesis of Bio-Antimicrobials with “Smart” Triiodides
Source: Molecules. 2021 Jun 10;26(12):3553. doi: 10.3390/molecules26123553 (PMC8230494; doi:10.3390/molecules26123553)

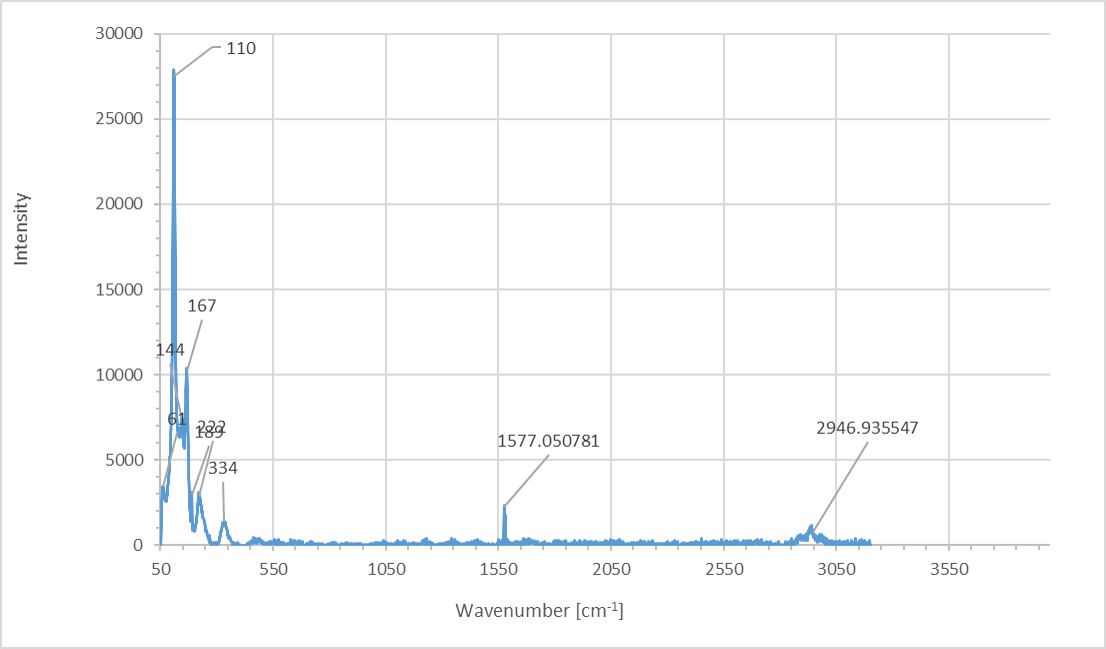

Supplement: Supplementary file 1 [file molecules-26-03553-s001.zip › molecules-1199471-supplementary/S1.jpg]

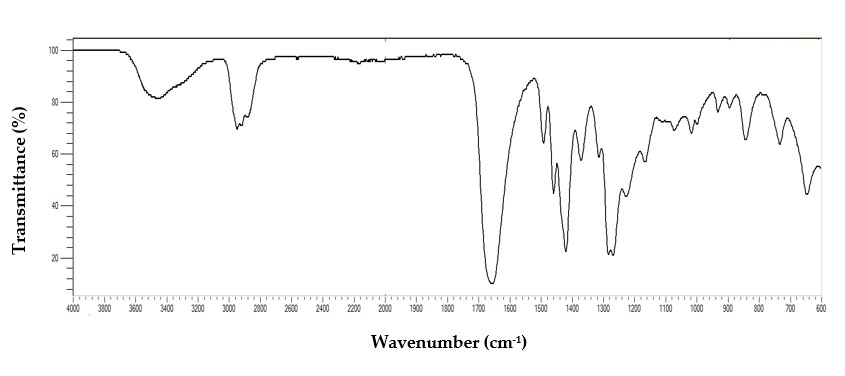

Supplement: Supplementary file 1 [file molecules-26-03553-s001.zip › molecules-1199471-supplementary/S2.jpg]

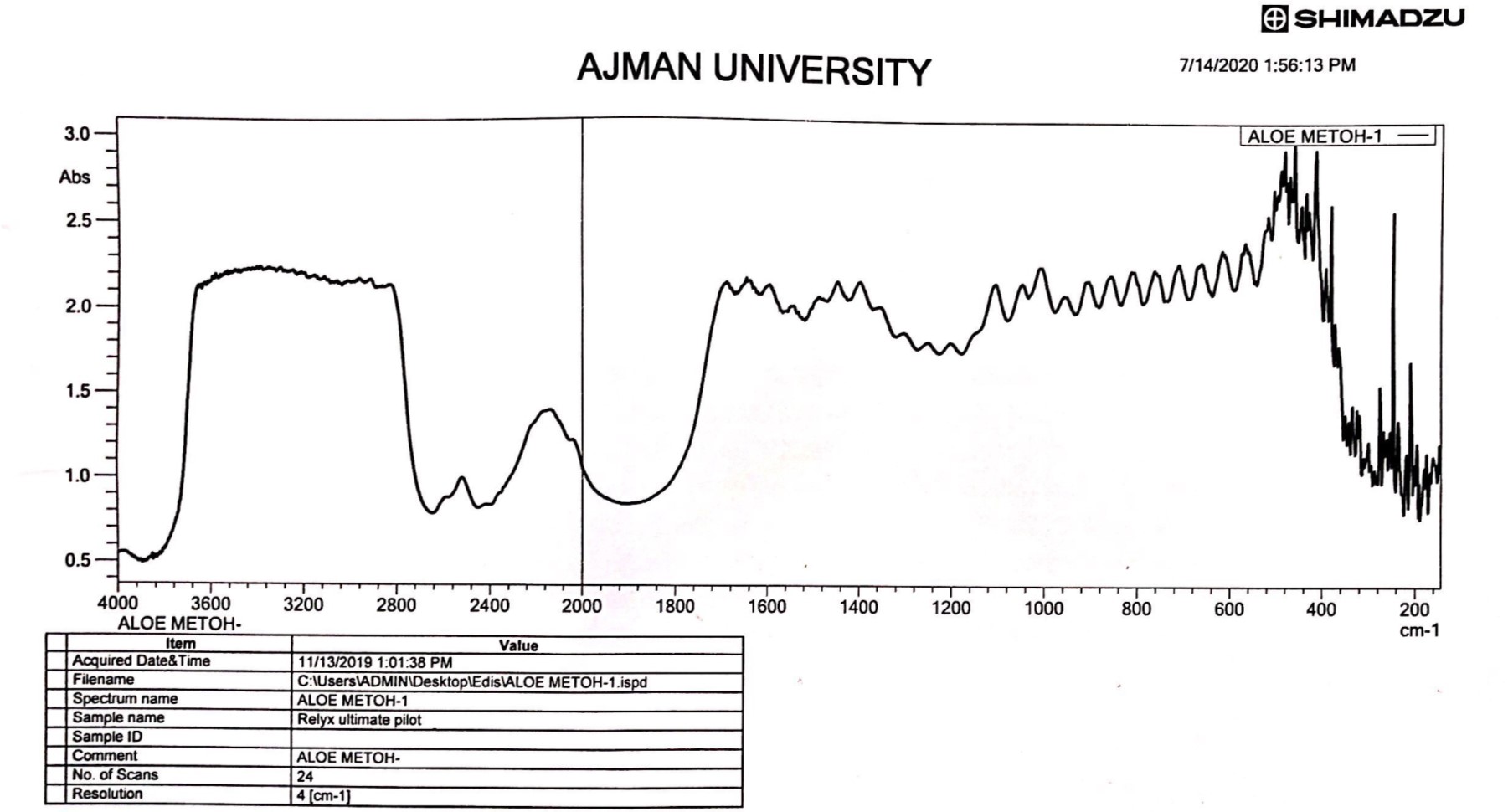

Supplement: Supplementary file 1 [file molecules-26-03553-s001.zip › molecules-1199471-supplementary/S3.jpg]
